# The role of biomaterials in the treatment of meniscal tears

**DOI:** 10.7717/peerj.4076

**Published:** 2017-11-17

**Authors:** Crystal O. Kean, Robert J. Brown, James Chapman

**Affiliations:** 1 School of Health, Medical and Applied Sciences, Central Queensland University, Rockhampton, Queensland, Australia; 2 Advanced Medical Solutions, Plymouth, UK

**Keywords:** Knee meniscus; biomaterials, Tissue engineering, Materials science, Scaffolds, Biomaterials

## Abstract

Extensive investigations over the recent decades have established the anatomical, biomechanical and functional importance of the meniscus in the knee joint. As a functioning part of the joint, it serves to prevent the deterioration of articular cartilage and subsequent osteoarthritis. To this end, meniscus repair and regeneration is of particular interest from the biomaterial, bioengineering and orthopaedic research community. Even though meniscal research is previously of a considerable volume, the research community with evolving material science, biology and medical advances are all pushing toward emerging novel solutions and approaches to the successful treatment of meniscal difficulties. This review presents a tactical evaluation of the latest biomaterials, experiments to simulate meniscal tears and the state-of-the-art materials and strategies currently used to treat tears.

## Introduction

The knee is considered a hinge joint; however, because it also features characteristics of an arthrodial joint, it is a more complex joint than other hinge joints such as the elbow and ankle. The knee consists of two articulations which form the tibiofemoral joint (further separated into the medial and lateral tibiofemoral joints) and the patellofemoral joint. The articulations are not entirely congruent and this arrangement allows for the combination of gliding and rolling motions which is constrained mainly by the ligaments of the knee. The menisci are fibrocartilagenous structures that sit on top of tibia to deepen the plateaus with the primary functions transmitting load through the joint and also serve to increase joint stability and lubrication of the articular cartilage (Seedhom, Dowson & Wright, 1974; Walker & Erkman, 1975; McDermott, Masouros & Amis, 2008).

The menisci are commonly injured due to traumatic events and/or degenerative stresses. In the United States alone, it was estimated that approximately 6.6 million patient visits to the emergency department between 1999 and 2008 were due to knee injuries equating to 2.29 knee injuries per 1,000 people (Gage et al., 2012; Reid et al., 2017). Furthermore, by 2060 the percentage of people reaching an age of 50 will reach 50% representing a change in population demographic and likelihood for pressures in the knee. A large proportion of knee injuries in the general population are meniscal related and meniscal injuries are even more common in a physically active population (Baker et al., 1985; Nielsen & Yde, 1991). Given the role meniscal tears, and subsequent partial or full removal of the meniscus, play in development of osteoarthritis (Englund, Roos & Lohmander, 2003; Roos et al., 1998) there is an increased interest in preservation of these structures following injury. For this reason, there is also an increased interest the role biomaterials play in meniscal repair, regeneration and replacement options.

Advances in materials technology have brought about an increased usage of biomaterials and medical devices in the body (Hallab, Link & McAfee, 2003; Chevalier, 2006; Yamamoto, Takagi & Ito, 2016). A biomaterial is a material or substance or combination of substances, other than drugs, synthetic or natural in origin, which can be used for any period of time (Bochyńska et al., 2016c; Brannigan & Dove , 2017; Kaur, 2017), which augments or replaces partially or totally any tissue, organ or function of the body in order to improve the quality of life of an individual (Bergmann & Stumpf, 2013). The biomaterial must be able to interact with the surrounding human tissue and body fluids to improve or replace the anatomical defect. Some examples of the recent advances for biomaterial use in medicine include knee and hip replacement (Walczak, Shahgaldi & Heatley, 1998; Bahraminasab & Farahmand, 2017), ocular implants (Lloyd, Faragher & Denyer, 2001; Baino et al., 2017; Mota et al., 2017), heart valves (Vongpatanasin, Hillis & Lange, 1996; Vander Roest & Merryman, 2016; Emmert & Hoerstrup, 2017), bone implants (Bròdano et al., 2014; Apicella et al., 2017), dental implants (Tamimi et al., 2014), biosensors (Sun et al., 2014; Calvo et al., 2017), orthopaedic screws and sutures (Waizy et al., 2014; Zhao et al., 2017) and tissue allografts (Cameron & Saha, 1997; Sanen et al., 2017). The achievements, in terms of biocompatibility, to lower risk of failure and improved surgical outcomes have contributed to the expanding use of biomaterials. For these reasons advancements in biomaterial development is and has been a significantly fast-growing area of research.

This review article will focus on providing a general review of the menisci and meniscal injuries. We also discuss biomaterials and the subsequent role biomaterials play in the surgical treatment options for meniscal repair, regeneration and replacement as well as future directions. While other reviews have been developed, their focus has been to provide an overview of materials only, this review provides significant detail on cell lines used, models and materials to support research momentum for future developmental medical breakthroughs.

## Meniscus

### Biomechanics and function

**Figure 1:**
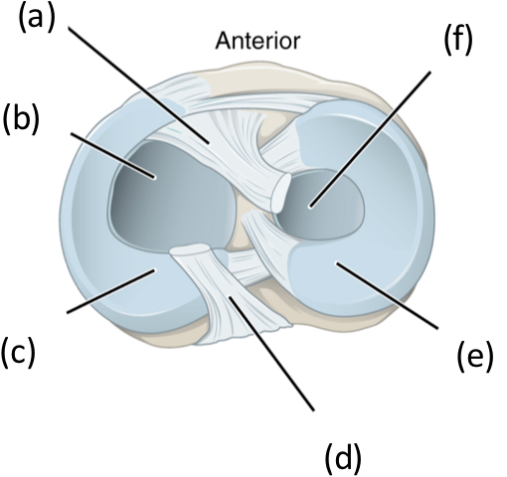
Superior view of the right tibia in the knee joint illustrating the menisci and cruciate ligaments. (A) anterior cruciate ligament, (B) articular cartilage on medial tibial condyle, (C) medial meniscus, (D) posterior cruciate ligament, (E) lateral meniscus, (F) articular cartilage on lateral tibial condyle.

The menisci are fibrocartilage structures, composed mainly of type 1 collagen, that sit on top of tibia, Fig. 1.

The lateral meniscus (e) is a C-shaped structure that covers approximately 80% of the lateral tibial plateau whereas the medial meniscus (c) is a U-shaped structure and covers only 60% of the medial tibial plateau. The menisci are relatively avascular with only 10–30% of the peripheral region of the medial meniscus and 10–25% of the lateral meniscus being vascular (Arnoczky & Warren, 1982). Based on its vascularisation, the menisci can be divided into three zones: the red-red vascular zone (outer peripheral region), the white-white avascular zone (inner region) and the red-white zone which lies between of the two other zones and has characteristics of other two zones. The red vascular region is thick and convex and attaches to the capsule of the joint whereas the white-white inner region is thin, concave and is a free edge unattached to the joint.

The menisci effectively deepen the tibial plateau and allow smooth articulation between the tibial and femoral condyles and the transmission of loads across the tibiofemoral joint. In full knee extension, the medial meniscus transmits approximately 50% of the load on the medial compartment, while lateral meniscus transmits approximately 70% of the load in the lateral compartment (Walker & Erkman, 1975). As knee flexion increases the amount of load transmitted to the lateral meniscus increases such that when the knee is flexed beyond 75°the entire load that passes through the lateral compartment, is transmitted by the lateral meniscus (Walker & Erkman, 1975). For the medial meniscus the increase in load transmission as the knee flexes is less apparent (Walker & Erkman, 1975). When the meniscus is intact, the load is well distributed across the tibiofemoral compartment; however when part or the entire meniscus is removed there is considerable alterations to load distribution such that there is a decrease in the contact area and increases in peak contact forces (Bedi et al., 2012; Lee et al., 2006; Ihn, Kim & Park, 1993).

### Meniscal tears

**Figure 2:**
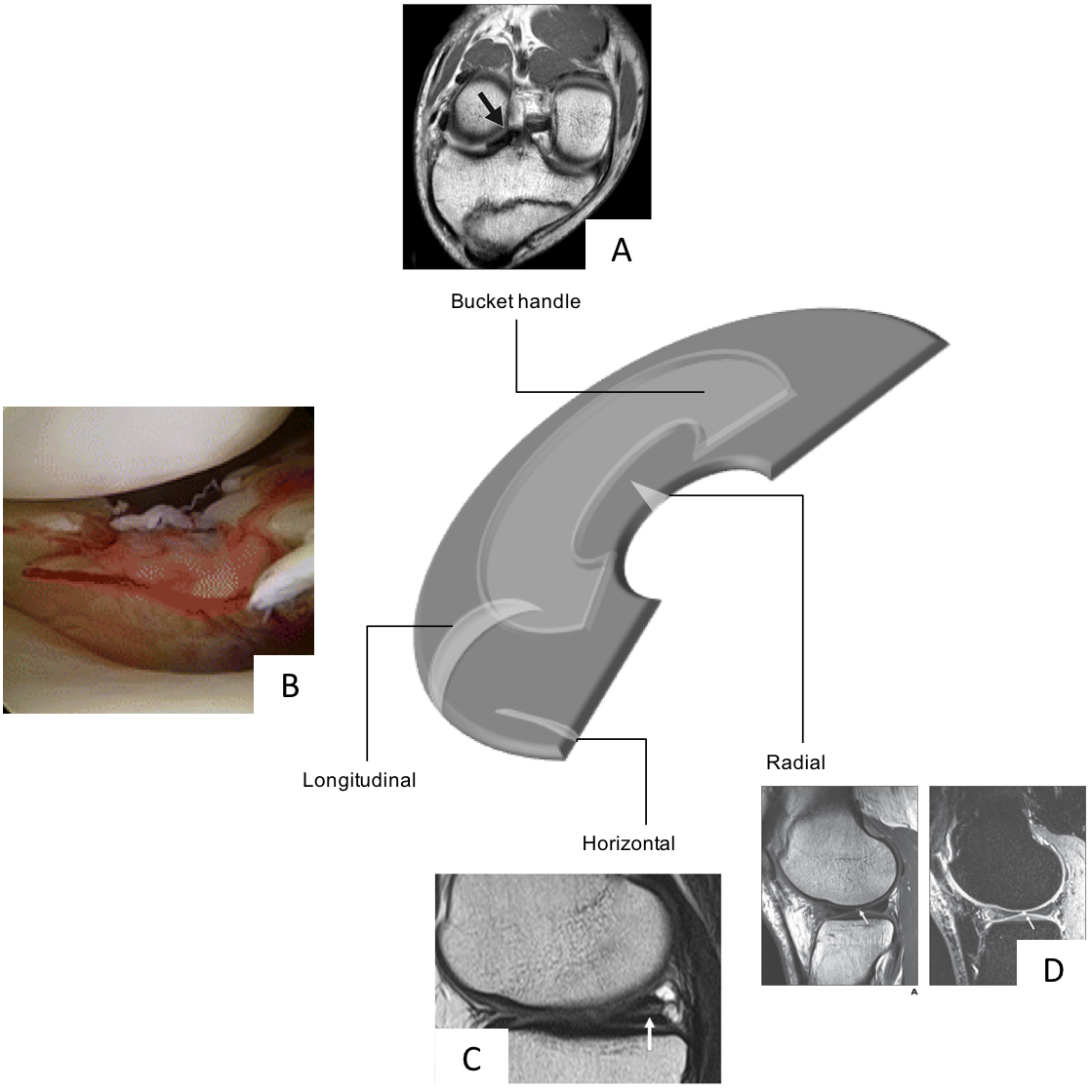
Schematic diagram highlighting the various types of meniscal tears, Bucket Handle MRI image taken from Han et al. (2015) (CC BY NC 3.0), Radial Tear, MRI image taken from Jung et al. (2012), and longitudinal (photograph taken from Feucht et al. (2015) (CC BY 4.0)) and horizontal tears (MRI taken from Ohishi et al. (2010) (CC BY 2.0)) all with permission.

Meniscal tears are one of the most common intra-articular knee injuries (Clayton & Court-Brown, 2008; Majewski, Susanne & Klaus, 2006) and is typically the result of an axial loading and rotational forces which result in a shear load on the meniscus (Browner, 2009). This may be a result of a traumatic event or cumulative stress leading to degenerative tears. The medial meniscus is more often injured than the lateral (Majewski, Susanne & Klaus, 2006); however, lateral meniscal tears are more often associated with acute ACL tear (Bellabarba, Bush-Joseph & Bach Jr, 1997). Although there is no uniformly accepted classification of meniscal tears, the classifications typically involve a description of the tear pattern and location. Common tear patterns that typically originate from traumatic events include longitudinal, bucket-handle, and radial tears (Greis et al., 2002). Whereas horizontal, flap and complex tears are typically seen in older adults and due to cumulative stress resulting in degeneration (Greis et al., 2002). The location of the tears may be classified based on the zone classification system purposed by Cooper, Arnoczky & Warren (1990) in which the menisci are divided into three radial zones (anterior, medial and posterior) and four circumferential zones (meniscosynovial junction or periphery, outer third, middle third and inner third of the menisci) (Fig. 2).

In a similar fashion to the zone classifications, tears may be graded as partial or full-thickness tears or using a grading scheme 0–III in which 0 indicates a normal intact menisci and III a full-thickness tear (Cooper, Arnoczky & Warren, 1990; Pihl et al., 2017).

### Available treatment options

Meniscal tears account for a significant portion of surgical procedures performed by orthopaedic surgeons, the patient experiences significant pain and sometimes complete disability from these tears and resulting procedures (Berthiaume et al., 2005). The surgical procedures involved in the treatment of a meniscal tear may include a partial or full meniscectomy or a meniscal repair. The meniscectomy procedures involve either part or all of the damaged meniscus being removed which in turn leads to higher rates of osteoarthritis in subsequent years. Surgical treatment of meniscal injuries has undergone a number of developments over the past two decades, moving from open arthroscopic surgery; from total to partial meniscectomy and adding novel treatments; such as repair using a variety of devices, materials, transplants, collagen implants or xenografts (Kim et al., 2013; Jiang et al., 2012; Grogan et al., 2017; Baek et al., 2017). If meniscectomy takes place or insisted upon, this procedure is mainly due to changes in load distribution across the articular cartilage as studies have shown that following total meniscectomy peak contact pressures increase by 253% and 165% following partial meniscectomy (Lee et al., 2006; Baratz, Fu & Mengato, 1986; Beamer et al., 2017; Van Egmond et al., 2017). Following meniscectomy, there is also evidence of reduced muscle strength, altered gait patterns and clinical outcomes (Hall et al., 2013; Hall et al., 2014; McLeod et al., 2012; Sturnieks et al., 2008a; Sturnieks, Besier & Lloyd, 2011; Sturnieks et al., 2008b; Salata, Gibbs & Sekiya, 2010; Scholes et al., 2017). For these reasons, there are an increasing number of interests in performing meniscal repair. What needs to be remembered is that not all meniscal tears are suitable for repair, and thus other treatment options such as meniscal replacement and regeneration are of considerable interest when a surgical intervention is necessary to improve any pain and symptoms.

## Biomaterials

Current treatment modalities for meniscal repair tears still carry their drawbacks and novel, robust and effective solutions are required. Some recent advances in meniscus research suggest that low cellularity, (King et al., 2017) dense ECM and poor vascularisation coupled with the inflammatory responses (King et al., 2017) in the knee joint are responsible for a lack of healing. Recently, biomaterials in the form of tissue adhesives have become available for clinical use: fibrin glue, (Bochyńska et al., 2016a) TissuGlu^®^, Dermabond^®^, (Balakrishnan et al., 2017) where the development of these new adhesive biomaterials has improved the properties of existing biomaterials alone (TissuGlu^®^, Raleigh, NC, USA; Ethicon Inc., Somerville, NJ, USA). Furthermore, these materials and strategies are not always a given success, presenting limitations to the accomplishment of the meniscal reparation.

### Tissue engineering using biomaterials

Of late, tissue-engineering and cellular biomaterial interactive concepts have been introduced to develop cellular-based reparation for cartilage regeneration (Temenoff & Mikos, 2000). The type of cell used to engineer cartilage is critical as a future goal of biomaterial development. Various cell populations that have been investigated for these roles include: chondrocytes (King et al., 2017; Chen & Cheng, 2006), mesenchymal stem cells, bone marrow stromal cells and perichondrocytes (Bruns et al., 1998). The choice of biomaterial is critical to the success of tissue engineering approaches for cartilage repair. The concept of ‘tissue engineering’ was first introduced and postulated by Green Jr (1977) where chondrocytes grown *ex vivo* could be transplanted into a region of tissue defect. Recently, tissue and biomaterial engineering concepts have been initiated to develop cellular based approaches for tissue repair (Freed et al., 1993). Typically, the process for engineering tissue involves the isolation of chondrocytes which are then seeded into a biocompatible matrix or scaffold and finally cultivated for implantation into the defected region. A large variety of biomaterials, natural and synthetic, have been employed as potential cell-carriers for tissue regeneration. The most common naturally occurring materials include type I and type II collagen-based biomaterials. Furthermore, some of the contrasting synthetic approaches include: polyglycolic acid or poly-L-lactic acid or other various composite mixtures (Chen & Cheng, 2006). In essence, an ideal candidate biomaterial would be a cell-carrier substance which closely mimics the natural environment in the surrounding matrix—as given by the definition of a biomaterial.

Regenerative approaches to meniscus repair occurs in a series of precise stages. It is typically understood that the low cellularity (endogenous meniscus cells and meniscus progenitors) (Mauck & Burdick, 2015), the dense ECM, poor vascularisation potential and the inflammatory responses typically linked to meniscus wounds all contribute to the success or failure of the meniscus healing and regeneration alone. This success of healing process is without a biomaterial introduced into the site. Based on these principles, the potential use of a biomaterial to develop and deliver a viable solution requires thought around this repair process.

Biomaterials are typically promoters of tissue repair through provision of scaffold layers for cellular attachment and growth and differentiation further acting as a vehicle for protein and gene transfer to regenerate functional tissue approaches (Chen, Zhang & Wu, 2010). Biomaterials in this area should have several properties to support *viable* repair. Typically, this is achieved through:

(1) The material must act as a support structure for cell lines (i.e., cells that are seeded *in vitro* are compatible, adhere to the material if required or certain cell lines are not required; filtered out). For meniscal repair the biomaterial must provide appropriate biomechanical functions after implantation to shield cells from damaging or compressive forces;
(2) Possess sufficient mechanical strength to protect the surrounding cells (cells should be mechanically stable i.e., cell attachment is maintained). For meniscal repair the biomaterial must maintain their shape and integrity, mechanical stability and strength for the defect area in question until new host tissue has been regenerated. Furthermore, it may be important to provide biological and mechanical context for cell differentiation, proliferation and attachment when a biomaterial is introduced into the knee. For example, it is now very well understood that cells are influenced by the local external environment including the adhesive and biophysical properties (Engler et al., 2006);
(3) Withstand *in vivo* forces during the joint movement operation (mechanical and structural stability of the biomaterial in the meniscus area needs to be able to withstand compressive and tensile forces (these forces have been aptly described in Paschos et al. (2017));
(4) Bioactivity should be provided to accommodate cellular attachment and cellular migration (the biomaterial in the meniscus will therefore be able to promote tissue regeneration). Furthermore, providing directional cues, such as chemotactic gradients to guide cells like endogenous cells to the injury site. Recently, some studies have shown that allowing migration of cells provides a motivation for the cells to attach and drives the cellular colonisation process (Mauck & Burdick, 2015; Greiner et al., 2014);
(5) The biomaterial should have biodegradable properties and be able to remodel as the novel cartilage grows, embeds and replaces the original construct; therefore, the matrix must be non-toxic, non-adhering and non-stimulating for inflammatory cells. The biomaterial for a meniscus should therefore facilitate host tissue integration and provide the appropriate biomechanical function in the knee.
(6) Furthermore, they should be non-immunogenic as this is catastrophic for the biomaterial insertion. For any biomaterial, this is important, to prevent rejection the appropriate level of biocompatibility and non-toxic ability needs to be considered.

### Biocompatibility

One of the most important non-mechanical requirements of orthopaedic biomaterials is biocompatibility. Biocompatibility is the ability of a substrate to exist in contact with tissues of the human body without causing an unacceptable degree of harm in the body. The biomaterial domain has been aptly described by Mardis and Kroeger, *“the utopian state where a biomaterial presents an interface with physiologic environment without the material adversely affecting the environment or environment adversely affecting the biomaterial”* (Mardis & Kroeger, 1988). An understanding of biocompatibility requires an appreciation of tissue cell, bacterial cell and host defence response to the insertion of a biomaterial in particular for this review—for meniscal interventions. Once the biomaterial has been placed into the body, a conditioning film containing biomolecules such as; water, electrolytes, cholesterol, vitamins, lipids and proteins (Chapman et al., 2013) (albumin, igG, fibronectin, fibrinogen, laminin, collagen and osteopontin) form on the surface long before cells are present and reach the state of equilibrium (Thevenot, Hu & Tang, 2008). In the very early implantation period or injury for this matter, inflammatory cells begin to proliferate, this is an immediate response (Anderson, Rodriguez & Chang, 2008). The first contact with tissue, proteins in blood and the interstitial fluids adsorb on to the biomaterial surface. An injury to vascularised connective tissue initiates the inflammatory response but also leads to the process of thrombus formation involving the activation of the extrinsic and also intrinsic coagulation, complement, fibrinolytic, kinin-generating systems and platelets (Anderson, Rodriguez & Chang, 2008). The conditioning layer represents a dynamic, ever-changing layer due to differential diffusion and mass transport of molecules in and out of the implant surface. Later stages of competitive binding then occur on the surface of the material owing to functional groups within the molecules. Cells therefore never see the ‘true’ surface of the biomaterial, but more correctly, respond and interact to a conditioned film that has consequently developed *in-situ*.

Following the conditioning sequence of the biomaterial, attachment cells secure themselves to the protein and protein matrices using integrin receptors. Thus, this conditioning layer is vital to the reaction of cells to the surface of the implanted biomaterial. The introduction of the biomaterial, the conditioning and immune response sequence is not always obvious as proteins have the ability to conform and expose epitopes that are not always identified as self-produced by the body’s immune system. Immune cells react as they detect what were once normal proteins and recognise them as foreign bodies. This process can result in a cascade of blood coagulation and chronic inflammation that can lead to occlusion of nutrients, changes in oxygen and fibrous capsule formation—operating toward total rejection by the body of the implanted biomaterial (Nasab & Hassan, 2010). The extent of the deformation process for proteins has been remedied based on the selection of material type. Surfaces are made more “passive” where chemical treatments are added to the manufacturing process. Passivation with acids such as nitric acid of stainless steel creates a less reactive oxide layer; this has been shown to improve the biocompatibility process. One added benefit to passivation is it serves as a means for removing foreign material from the surface, such as bacteria or biofilms (Blumenfeld & Bargar, 2006). Passivation can also be used to surface-modify natural or synthetic polymer biomaterial substrates for meniscal tear applications. For example, albumin, where the resulting surface passivation has been shown to reduce and prevent clotting (Kaur, 2017; Hanker & Giammara, 1988).

### Role of biomaterials in meniscal repair

An article by Abrams et al. (2013) has shown that while there was no increase in the overall number of meniscal procedures, over a seven-year period there has been an 11.4% increase in isolated meniscal repairs and a 48.3% increase in meniscal repairs in combination with ACL reconstruction. This sharp increase in meniscal repair treatment is mainly due to the increased knowledge in the importance of the preservation of the meniscus to maintain normal knee function and prevent osteoarthritis. It has been shown that following meniscal repair, peak contact pressures are similar to that experienced with an intact meniscus (Bedi et al., 2010). Unfortunately, it is estimated that currently only 20% of all meniscal tears are repairable. Tears in the meniscal periphery (i.e., the red-red vascular zone) are most likely to heal whereas those in the meniscal avascular zone (i.e., the white-white zone) are unlikely to heal and those in the red-white zone have the potential to heal (Belzer & Cannon, 1993; Noyes & Barber-Westin, 2012). Besides vascularisation, tear type and various patient characteristics can influence decision making on treatment options and success of a meniscal repair. Typically, tears that are less than 2 cm in length, longitudinal and acute are more amendable to repair than larger tears (Taylor & Rodeo, 2013; Laible, Stein & Kiridly, 2013). Meniscal repairs are also not typically recommended for degenerative tears and thus repair success is typically superior in young patients (less than 50 years of age) (Laible, Stein & Kiridly, 2013). When appropriately performed, meniscal repairs provide considerable improvements in terms of clinical outcome and osteoarthritis prevention compared to a partial meniscectomy (Stein et al., 2010). Thus, finding ways to increase the number of meniscal tears that can be treated by meniscal reparation is of great importance.

Vascularisation in the meniscus tissue is of high relevance to biomaterial design. From prenatal development up until after birth, the meniscus is fully vascularised. Following this, from the age of ten, vascularisation reduces to 30% of the meniscus and at maturity the meniscus only in the peripheral region of approximately 10% of the tissue. Vascularisation represents another challenge in meeting the requirements of success for biomaterial implantation as a meniscus operation. Vascular endothelial growth factor enhances the blood vessel density in peri-implant spaces. Biomaterial scaffolds of knee menisci exist in a highly challenging environment as little vascular support is provided in this region of the body. Electrospinning of polymeric fibres can be produced to support other engineering applications such as blood vessel, tendons, meniscus and cartilage (Xu et al., 2004). Some authors have used unique biodegradable nanofibers as a scaffold to support blood vessel engineering. They have demonstrated that fibres of 500 nm with an aligned topography is able to mimic the circumferential orientation of cells and fibrils (Leong et al., 2009). The authors have postulated that macrophage within the CES produce angiogeneic growth factors that potentially stimulates vascularisation.

### Role of biomaterials in meniscal replacement/regeneration

Owing to the limited percentage of meniscal tears that can be repaired and the poor clinical results with untreated symptomatic meniscal injuries and partial meniscectomy, biomaterial synthetic and allogeneic (genetically dissimilar) interacting biomaterials have been investigated to serve as a matrix to lead meniscal regeneration medicine, particularly as a cellular support.

#### Hydrogels

Using a biomaterial that has the ability to seamlessly integrate in to water matrices is another attractive property in regenerative medicine applications for meniscal repair. Hydrogels are one such material with a considerable water based content; using hydrated polymer networks capable of absorbing and retaining fluids. Hydrogels are determined by their monomeric composition, crosslinking density and polymerisation ability. Due to the crosslinking chemistry the polymer remains insoluble in solution. The insolubility, along with the high hydration threshold, make hydrogels appealing to use for human tissue mimetics (Kobayashi, Chang & Oka, 2005). As an example, some authors have used a poly (vinyl alcohol) hydrogel with a water content of approximately 90% to produce knee implants using a rabbit model (Makris, Hadidi & Athanasiou, 2011). The implant replaced the whole lateral meniscus over two years. In a subsequent study, the hydrogel implant was not able to prevent damage to articular cartilage but was able to reduce progression of meniscal decay. Some of the new and emerging biomaterial types have been shown in Table 1.

**Table 1:** Summary of biomaterial studies used in meniscus research.

Biomaterial used	Author	Engineering region	Success(es)	Species model	Ramifications
Synthetic polymers					
Hydrogels	Kim & Healy (2003)	Meniscus tissue engineering	Maintained 90% water content that are not degraded by proteases. The hydrogels used in this study were incorporated with non-reducible collagen crosslink, pyridinoline.	Mammalian	Peptide linked hydrogels have the ability to be tailored to create environment responsive artificial extracellular matrices that are degraded by proteases.
	Rey-Rico, Cucchiarini & Madry (2017)	Meniscus tissue engineering	Review article providing results on specific 3D microenvironments using hydrogels. Many hydrogel polymers were used in this paper.	Human and animal models	Hydrogels can be used as a platform for precision and targeted meniscus tissue engineering
Polygolic acid	Buma et al. (2004)	Meniscus tissue repair	Optimal pore geometry realised (15–25 µm)	Canine	Autologous meniscus cells seem to be the optimal cell source for tissue engineering. Research should be stimulated to demonstrate suitability of other cell lines for meniscal repair.
	Ibarra et al. (1997)	Tissue engineered meniscal tissue repair	Used to replace massive tears or completely resected menisci	Bovine	A pivotal paper to show that autologous cells could eventually be used to replace allografts for meniscus transplantation.
Natural Polymers					
Collagen-glycosaminoglycan (GAG)	Mueller et al. (1999)	Regeneration approaches to knee meniscus	Type II GAG matrix increased DNA content and cellular response to the matrix over 3 weeks	Canine	Type II matrix for the number of cells and the higher GAG synthesis of type II matrices commend further investigation and regeneration of meniscus *in vivo*.
	McCorry & Bonassar (2017)	Tissue engineering for meniscal repair	Mesenchymel stem cells increased the GAG and collagen production in both co-culture and monoculture groups in a 4 week study.	Bovine	MSC lacks fibre organisation capability. The study suggests that GAG production and fibre formation are largely linked, therefore co-culture techniques can be used to balance synthetic properties and matrix modelling capability.
Collagen sponge	Walsh et al. (1999)	Medial meniscus repair	Collagen sponge acted as a scaffold producing abundant tissue repair.	Canine	Degenerative changes were present in both groups indicating biomechanical function was compromised.
	Murakami et al. (2017)	Meniscal scaffold structure and repair	Collagen sponges demonstrated greater strength. At 12 weeks stress and compression testing was performed, lower inflammation was noted in all samples coated with PGA. Foreign body multinucleated giant cells in implanted groups appeared in weeks 8.	Lapine	Meniscal scaffolds using PGA should possess biological and biomechanical functions. The PGA coating was a beneficial property of the scaffold and offers excellent biomechanical function, regeneration and ultimately less inflammation in this material type.
Copolymeric (L-lactide/epsilon-caprolactone)	De Groot et al. (1997)	Meniscal repair	Copolymer implants demonstrated improved adhesion; fibrocartilage was affected of the compression modulus. The copolymer was degradable.	Canine	Tearing problems usually associated with sutures were partly circumnavigated, this paper paves the way for more work in meniscal prostheses including transplantation.

#### Chitin

Chitin sutures are an emerging material of choice for improvement of the mechanical properties of a knee healing process (Brittberg et al., 1994). Owing to its favourable mechanical properties, chitin has been used for applications that require exceptional integrity and physical strength in surgical sutures, some new medical textiles and even as bone substitute materials.

#### Nanofibres

Electrospun scaffolds are also another emerging biomaterial that has begun to be used for cellular adhesion applications in regenerative medicine. The fibres have the ability to mimic both anisotropy of fibrous tissues and withstand high load forces that are imposed on the tissue during physiological motions (Ionescu & Mauck, 2012). The electrospun biomaterials can also be tailored to produce various size, shapes and makeup (for example coaxial materials) will influence cell interactions and the cells will begin to proliferate and adhere and finally deposit matrix on to the fibre network. These interactions provide improved mechanical properties for the biomaterial scaffold over time. Fibres can be collected on to rotating drums or flat collection plates, depending on the order, orientation and architectures that they are required. Cells typically are seeded on to these scaffolds and cultured over time *in vitro*. In a study by Passaretti et al. (2001) tensile modulus was seen to improve on fibre aligned scaffolds some 7-fold higher than disorganised fibres approaching the value of a normal meniscus. Essentially, the authors determined that cells prefer to align on ordered scaffold fibres rather than disorganised arrangements. Further to these findings, internal organisation in the form of sheet fibres can also be arranged for tissue-mimicking structures. Specifically, for meniscal tissue engineering, cells can be isolated, expanded and manually seeded on to the surfaces of electrospun scaffolds prior to an implantation operation, expediting the regenerative process. Cells along with host cells will migrate on to the newly implanted scaffold and deposit proteoglycan and collagen. Some implantation methods require surgery prior to this implant step to isolate the cells prior to seeding, maturation and implantation.

#### Biodegradable polymers

Some of the more current treatment methods for repair of meniscal tears are somewhat indifferent for positive results and outcomes. Tissue adhesives are a promising alternative, owing to their ease of application and minimal tissue trauma. Co-polymeric tissue adhesives have been shown to have adhesive strengths of 40–50 kPa and hold edges of meniscal tears together during healing periods. These results indicate that copolymers are able to improve tissue capacity for self-repair specific for meniscal applications. Other authors have used amphiphilic copolymers based on polyethylene glycol, trimethylene carbonate and citric acid to synthesise end-functionalised hexamethylene diisocyanate to form biodegradable hyper-branched tissue adhesives. The work showcases resorbable tissue materials for meniscus repair. The materials have excellent mechanical and adhesive properties that could be adjusted through variation of the composition of the copolymers (Bochyńska et al., 2016c). Regenerative engineering converges a number of research areas and is truly multidisciplinary inclusive of tissue engineering, advanced materials, stem cell science and developmental biology to regenerate complex tissues from menisci to whole limbs (Narayanan et al., 2016). Clinical applications of tissue engineering technologies are still relatively restricted owing in part to the limited number of biomaterials that are approved for human use. While many biomaterials have been developed, their translation into practice has been extremely slow. Consequently, many researchers are still using biodegradable choices that were approved some 30 years ago. Most degradable biomaterials used to date comprise of synthetic polyesters:

• Poly(L-lactic acid) PLLA;
• Poly(L–glyolic acid) PLGA; and
• Biological polymers such as: alginate or chitosan, collagen or fibrin (Middleton & Tipton, 2000).

Polyester-based polymers are clearly an excellent candidate as a synthetic biodegradable and bio-absorbable material for medical applications. The use of synthetic polyesters as biomaterials allow the unique control of the morphology, mechanical properties and degradation profiles measured through the monomer selection, polymer composition informed through the copolymer and homopolymer, stereo-complexation and also the molecular weight. In an excellent review by Brannigan and Dove, degradation mechanisms has been discussed in detail, in a clinical research capacity—these parameters are of paramount importance to understand the behaviour of the material *in vitro* or *in vivo*. The authors discuss enzymatic, oxidative, and physical degradation. Brannigan & Dove (2017) discuss the use and importance of polyester type Poly-HDPE scaffolds with an interconnected porous structure for cartilage regeneration. In their work, neocartilage formation within a synthetic polyester scaffold based on polymerisation of high internal phase emulsions were used. The fabrication of polyHIPE polymers (PHP) was ordered to have highly porous giving structure to the cartilage with a higher potential in force wear. Another example of the use of biodegradable polymers in meniscal repair research includes, poly lactic acid or L-PLA is used in menisca reconstruction in a study using canines, the presence of macrophages, fibroblasts, giant cells and lymphocytes were observed to be attaching to the material. From this study it seems that biocompatibility reduces when the degradation process ensues. This degradation property therefore promoted inflammatory responses and thus rejection (Jones et al., 2002).

### Discovery of new biomaterials—beyond state of the art

The next phase in developing knee meniscal biomaterials for replacement and or regeneration applications extends to the design, discovery and evaluation of bioactive materials.

Bioactive meniscal materials have been used with some significantly exciting and promising results. For example, bioactive scaffolds have been shown to modulate local ECM density to improve repair (Shin, Jo & Mikos, 2003). A novel biphasic collagen scaffold and shown to support meniscal repair *in vivo* to support meniscal cell ingrowth but also producing ECM *in vitro* by Howard et al. (2016). The authors have shown that the addition of PRP enhanced scaffold enhanced healing (Howard et al., 2016). Other emerging materials which could show potential in meniscal repair include: cartilage matrix is also a promising material for cartilage regeneration given the emerging evidence supporting its chondroinductive character. The cartilage matrix is a promising material for hyaline cartilage tissue engineering applications and has been shown that cell derived matrix and ECM materials and have been demonstrated to show established decellularisation, representing an excellent and promising choice of new material for future direction. (Redman, Oldfield & Archer, 2005). A drawback so far is that the FDA regulatory approval may affect the decision to use a native or cell-derived matrix. To expedite FDA approval, a full chemical decellularisation of allogeneic matrix may be used—this way, removal of cells ensures no cross-species interactions (Sutherland et al., 2015). For example, allogeneic cells from bone marrow can be used in cardiac repair (Lemcke et al., 2017).

Initially, this is a relatively straightforward process whereby advanced synthesis of new materials can be performed. The difficulty lies with producing the novel activity and evaluation of the behaviour of the material in the biological system. Adapting the surface properties through the addition of synthetic peptides and or molecular drugs can yield thousands of candidate materials for testing. This approach has already been realised in the form of library derived screening techniques using commercially available methacrylate monomers—influencing attachment, growth, proliferation and differentiation of human embryonic stem cells (Anderson, Levenberg & Langer, 2004).

Further developments in biomaterials will continue to expand at the interface of nanotechnology. Understanding the tribological interaction with the surrounding interface of the human body is an approach that is being realised using the “bottom-up” approach (Zur et al., 2011). The bottom up approach will develop novel, self-assembling and environment reactive biomaterials. In particular, self-assembling peptides offer a new approach owing to the large variety of sequences that can be produced by chemical synthesis. These advances include the design of short peptides that have the ability to resemble nanofilaments which are compatible *in vitro*, without rejection. The use of peptides in polymeric materials allows for resistance in concentration, pH or level of divalent cation variability (Hartgerink, Beniash & Stupp, 2002).

The use of combinational gene therapy and biomaterial approaches is a recent technique to remedy meniscal lesions formed when orthopaedic surgery and loss of the meniscus has accelerated in the patient. The lack of therapeutic options suggests there is a need for improved treatments to enhance meniscal tear repair treatments/operations. Combinational approaches may also provide strategies to support this remedy (Cucchiarini et al., 2016). Gene therapy, can be directly applied as a combination or direct approach to meniscal repair strategies. A recent evaluation on gene therapy with cell and tissue engineering-based approaches demonstrates a six strategy approach: (a) directly using gene transfer vectors (Elsler et al., 2012), (b) administering genetically modified cells (Nakagawa et al., 2015), which could be fraught upon in some researching countries, continents, (c) implanting the biocompatible material that can deliver the recombinant factor, as we have seen rejection may be a potential problem with this result, (d) applying autologous platelet-rich plasma or fibrin clotting factors, (e) providing a biomaterial that delivers a gene transfer vector, (f) transplanting a material seeded with cells, again, we envisage a potential rejection with this treatment.

#### Stem cell approaches

Exciting new techniques are emerging as non-invasive approaches to meniscal tear correction using stem cells. The promising use of new tissue engineering approaches have incorporated natural biomaterials made from extracellular matrices of decellularised tissues from the heart, lung and bone for example (Yuan et al., 2017). The use of a scaffold or ‘shell’ to align stem cells upon in a given feature is fast becoming attractive. Decullularisation preserves the molecular composition with tissue specific molecules including structural and mechanical features present in the original tissue. The preservation step will aid in guiding the behaviour of the therapeutic cells and facilitate tissue development when implanted, non-invasively to the meniscal tear region.

*In vitro* studies have also been used to investigate tissue surface modification with collagenase to prime the surface where the addition of the TGF-beta3 cells has been proven to increase the number of cells present in meniscal tears repaired with newly developed tissue adhesives such as isocyanate-terminated block polymers. For example, Bochynske et al. have used cylindrical explants harvested from bovine menisci, the explants were simulated to possesses a full thickness-tear where the explants were then removed and glued back to the defect. In addition, the repair constructs were then culture with and without the addition of TGF-beta3 and assessed for their histological appearances. The histological staining of the constructs confirmed that cytotoxicity was not an issue and after 28 days, meniscal cells were present in the contact glues (Bochyńska et al., 2016b). The results demonstrate that the use of TGF-beta 3 induces thicker cell numbers round the edges of the annulus of the explants and also appears to be a promising treatment for tears using these glue types.

#### Biomimetics

One final, prominent field emerging in material science lies with biomimetic biomaterial approaches (Chapman et al., 2014). Biomimetic materials are materials that have been directly replicated from nature to produce a solution to a specific problem. Some synthetic polymers may be able to provide a more biomimetic environment than the previously discussed hydrogel approach. Functionalising hydrogels using chemistry is one strategy that requires future investigation. Hydrogels have the ability to create a more ‘native’ microenvironment for cells in a particular area of the body—i.e., the knee. For example, scaffolds with biomimetics have been developed for tissue engineering based on a multidisciplinary approach using engineering of biomaterials and nano/micro structuring of the defect tissues. The use of 3D bioprinting is considered to be conventional however, the technique has allowed for traditional fabrication methods for porous bone and cartilage regeneration to be taken in new directions using gas forming, soluble particle leaching or freeze drying. Newer methods to generate porous scaffolds using biodegradable polymers include using gas forming of porogens (ammonium bicarbonate particles). Injectable hydrogels using click chemistry (high yielding, wide in scope molecules) have also shown to be highly advantageous for local delivery of bioactive molecules, ease of handing and reduced invasiveness, these techniques have been demonstrated to be potentially used in 3D bioprinting (Jo, Kim & Noh, 2012). The use of hand held 3D matrix printing using a bio-pen has allowed for in-situ printing and repair to take place. This will be a major development in regenerative medicine (Di Bella et al., 2017). The most recent and emerging areas for biomimetic medical materials are (Chen et al., 2016): (1) 3D bioprinting (focussed on medical materials); (2) designing nano/micro technologies; (3) surface modification of biomaterials for their cellular interaction ability; (4) clinical aspects of biomaterials for cartilage focussing on cells, scaffolds and cytokines (Fratzl & Weinkamer, 2007). The traditional methods still have many advantages (Chen et al., 2016), but as 3D printing techniques develop coupled with new developments in chemistry of the biomaterial, the use of biomimetic design and the inherent properties linked to biocompatibility will enable more advanced developments in the future of meniscal repair.

## Conclusions

Evidently, the diversity of biomaterials for meniscal applications is immense. Many approaches to mimicking the structure and function of the ECM have been conceived. It is crucial that these advances continue to be investigated for their ability to interact within a biological system. As biomaterials advance and new methods of delivery develop, inclusive of minimal invasive surgery move forward—the field of meniscal tears and treatment will be greatly advanced and if not greatly reduced in the coming decade.

## Additional Information and Declarations

### Data Availability

The following information was supplied regarding data availability: No raw code exists for this work.
